# Semiempirical modeling of the effects of the intrinsic and extrinsic optical phonons on the performance of the graphene-based devices

**DOI:** 10.1038/s41598-022-14073-5

**Published:** 2022-06-21

**Authors:** Sharare Jalalvandi, Sara Darbari, Mohammad Kazem Moravvej-Farshi

**Affiliations:** Nano Plasmo-Photonic Research Group, Faculty of Electrical and Computer Engineering, Tarbiat Modares University, Tehran, 1411713116 Iran

**Keywords:** Electronic properties and devices, Optical properties and devices

## Abstract

Surface plasmons in graphene have mainly been affected by intrinsic optical phonons due to the vibrations of the carbon atoms and surface polar optical phonons (S-POPs) of the underlying dielectric surface. This plasmon hybridization dramatically changes the features of the plasmonic devices. However, a complete theoretical model for the graphene impedance to consider the optical phonons effects is yet remained to be developed. Here, we show how to derive a model for graphene impedance to include such impacts on graphene surface plasmons. Earlier models suffer from two limitations—i.e., the inability to show (i) the transformation of a single pure plasmonic mode into multiple hybrid plasmon–phonon excitations and (ii) the damping effect for energies beyond that of the intrinsic optical phonons due to the phonon emission. Our new model overcomes these two limitations. Then, we calculate the extinction spectra for a one-dimensional periodic array of graphene ribbons obtained through the impedance boundary condition method, addressing these obstacles. These spectra are directly related to graphene impedance, modeled using the dielectric function we developed in our earlier work. The extinction spectra show the presented model overcoming the limitations, firmly fitting the experimental data reported by others. Furthermore, we introduce our developed model for graphene to the CST Studio software to verify the accuracy of our extinction relation and impedance model. This study can be a step forward correctly predicting the behavior of graphene-based plasmonic devices.

## Introduction

Electron–phonon interaction is an inevitable source of scattering, strongly affecting carrier transport^1–4^. In particular, plasmon dispersion in graphene has been altered significantly by intrinsic and extrinsic optical phonons exposure^5–11^. Plasmons with energies higher than intrinsic optical phonons (*≈* 0.2 eV) damp through inherent phonon emission into the intraband single-particle excitation region^12,13^. Furthermore, the extrinsic polar optical phonons of the surface (i.e., S-POPs) of the underlying polar-dielectric substrate significantly affect the dispersion of the surface plasmons of graphene. This interaction turns the primary plasmonic mode into several hybrid plasmon–phonon modes, depending on the number of S-POP excitation modes on the substrate surface, as we have demonstrated earlier^5^.

Electron–phonon interactions enhance the graphene impedance, altering its extinction spectra—i.e., the amount of radiation lost by a sample as a function of frequency. In other words, electron scatterings result in loss of the energy and momentum of the carrier and hence, hinder the electronic response to an applied electric field due to the impedance enhancement. The scattering rate is directly associated with the number of optical phonons. Hence, to examine the impedance properly, the impact of the atomic vibrations must be considered, particularly at room temperature when the number of phonons is high enough^6^. The existing resistance formulas for graphene proposed earlier^14,15^, obtained through semi-classical models, taking interband and intraband transitions into account and calculated electrical conductivity. Since these models neither include the impact of intrinsic phonons, nor that of extrinsic S-POPs, they suffer from two principal limitations. They can neither predict the plasmon damping phenomenon due to intrinsic optical phonon emission—i.e., demonstrated experimentally^12–14,16–19^, nor foresee the impacts of the extraneous S-POPs on the plasmon excitations for graphene loaded on polar dielectrics^13,19^. A substrate like SiC, hBN, or SiO_2_, with one or more (*n*) dominant vibrational S-POPs modes, splits a single graphene plasmonic mode into *n* + 1 hybrid plasmon–phonon modes. This effect is prevalent near the splitting energies, becoming negligible far from those values^5,13^. So long as the working frequency is near the phonon’s frequency, we should worry about the correctness of the impedance model in predicting the exact output extinction ratio. Notice, the semi-classical spatial dispersion model tolerable results for frequencies far from vibrational excitations in which the phonon effects are unimportant.

This work aims to overcome these discrepancies by introducing a new model for graphene impedance, taking the presence of intrinsic optical phonons and extrinsic S-POPs into account. In general, the impedance is directly related to two values: (i) The loss function, obtained by the imaginary part of the dielectric function, can be developed by random phase approximation (RPA)—i.e., Im{ε_RPA_}^−1^;^5,13,19^ (ii) The number of intrinsic optical phonons and extrinsic S-POPs. Our accurate and straightforward model includes these parameters. We also acquire an exact relation for evaluating extinction values, benefiting from the impedance boundary condition method, showing an excellent agreement between theory and experiment by utilizing the modeled impedance. Our new model indeed predicts the damping of hybrid modes for energies higher than that of the intrinsic optical phonons and the mode splitting caused by the extrinsic S-POPs. Moreover, we define our impedance model for graphene in CST Microwave Studio software and obtain the desired extinction values. One of the main limitations of the built-in graphene model in CST is that it is written based on the intraband and interband equations and does not include the effects of vibrational modes. However, we modify the model to the impacts of intrinsic and extrinsic phonons. The results show that the presented formulas work rightly, paving the way for accurate prediction of the experimental results.

Different approaches are employed to excite plasmons in graphene, such as patterning into one or two-dimensional (1D or 2D) periodic arrays, like ribbons and discs, light scattering from structures adjacent to graphene, or coupling to a grating placed on top or below the graphene. The first approach is the most straightforward based on today’s technological progress. In this approach, by adjusting parameters like periodicity, width, or the spaces between nearby arrays, one can quickly obtain desired plasmonic peak frequency^19^. Here, we take in a 1D periodic array of infinitely long (in the *y*-direction) graphene ribbons of width *w*_x_ and periodicity *d*_*x*_ (along the *x*-direction). Moreover, we obtain an extinction relation when a normal s-polarized (i.e., polarization perpendicular to the ribbons) lightwave illuminates periodic plasmonic patterns, exciting surface plasmons on the graphene surface. A p-polarized light (i.e., polarization parallel to the stripes) cannot recognize the periodicity and hence does not excite surface plasmons^19^. We use the impedance boundary condition method to reach the desired extinction relation and apply the RPA dielectric function to introduce desired impedance.

The organization of the rest of the paper is as follows. In the next section, after a brief introduction of Green’s function for an array of ribbons (i.e., the potential response produced by line sources generating the stripes), we obtain the extinction ratio formula. Then, we model the graphene impedance in the presence of the optical phonons. Next, we present the numerical results, including extinction spectra of two periodic plasmonic arrays, one placed on a diamond-like-carbon (DLC) substrate—i.e., non-polar substrate—and the other on SiO_2_ as a polar substrate with three dominant vibrating modes. Then, we compare the results of our theoretical model with the existing experimental results, showing perfect agreement among them. Moreover, we present the outcomes of software simulations to demonstrate our model’s correctness. The material introduced in CST software comes in Supplementary Information. Finally, we complete the paper with conclusive remarks.

## Theory

**Figure 1:**
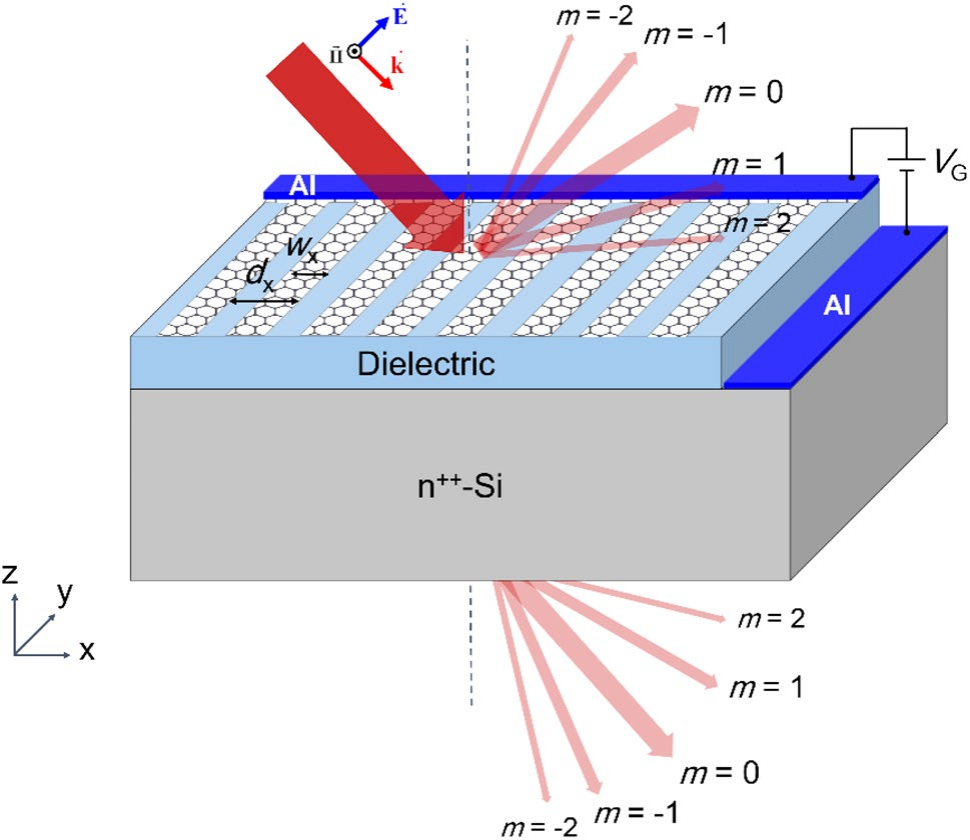
Schematic of a typical structure containing a 1D periodic array of graphene ribbons of width *w*_*x*_ and periodicity *d*_*x*_ placed on a dielectric grown on top of a Si substrate. The incident, scattered, and transmitted wave orders, *m*, are also shown by red arrows.

Figure 1 shows the schematic of a typical structure containing a 1D periodic array of graphene ribbons of width *w*_x_ and periodicity *d*_x_, placed on a nonpolar (polar) dielectric grown on an n^++^-Si together with the aluminum layer, acting as the ohmic contact to the gate material (the dielectric layer). As observed in the figure, by applying an appropriate external bias (*V*_G_) between the gate terminal and the aluminum contact on graphene, interconnecting the graphene ribbons, one can control the graphene chemical potential, *μ*_C_. Letters *m* label the different orders of the transmitted and scattered waves in arbitrary directions depicted by red arrows.

### Extinction relation

Green’s function can represent the solution of Maxwell’s equations in 1D periodic structures, such as periodically arrayed graphene ribbons, considering a line source shifted by an appropriate phase^20^,

1 $$G_{{{\text{1D}}}} \left( {\left. {x,z} \right|x^{\prime},z^{\prime}} \right) = \sum\limits_{m = - \infty }^{\infty } {\frac{{e^{{ - jk_{zm} \left| {z - z^{\prime}} \right|}} }}{{j2dk_{zm} }}e^{{jk_{xm} \left( {x - x^{\prime}} \right)}} } ,$$

where (*x*′, *z*′) introduces *y*-expanded line source position, *m* is the scattered waves’ order, *k*_xm(zm)_ is the wavenumbers along the *x*(*z*)-axis,

2a $$k_{{{\text{xm}}}} = k_{x} - \frac{2m\pi }{{d_{{\text{x}}} }},$$

and

2b $$k_{{{\text{zm}}}} = \sqrt {k_{0}^{2} - k_{{{\text{xm}}}}^{2} - k_{y}^{2} } .$$

In (2a) and (2b), *k*_*x*(*y*)_ and *k*_0_ = 2π/λ represent the *x*(*y*)-component of the incident wavevector and the related wavenumber, with wavelength λ. Under a normal s-polarized illumination *k*_*x*(*y*)_ = 0. So, according to 2(b), for *k*_xm_ > *k*_0_, *k*_zm_ becomes imaginary, degrading the Green’s function rapidly in the far-field region, z ≫ z′, making the reflected and transmitted lights invisible. For the sub-wavelength unit cell, the condition of *k*_xm_ > *k*_0_ is valid only for *m* ≠ 0, meaning that only the zeroth-order scattered light can propagate^20^. Assume an array is positioned in an *x–y* plane at *z*′ = 0, ignore the terms *m* ≠ 0, and use the impedance boundary condition method^20^ to reach the extinction expression for the 1D array,

3 $$\alpha_{{{\text{1D}}}} = 1 - \left| {\frac{{\eta_{0} \left( {\eta_{0} + 2Z_{{\text{G,s}}} } \right)}}{{\eta_{0}^{2} + 2\eta_{0} \left( {Z_{{\text{G,s}}} + Z_{{\text{G,p}}} } \right) + 4Z_{{\text{G,s}}} Z_{{\text{G,p}}} }}} \right|,$$

where *η*_*0*_ = 376.73 is the free space impedance and *Z*_G,s(p)_ represents the impedance of a 1D periodic graphene array illuminated by an *s*(*p*)-polarized wave^21^

4a $$Z_{{{\text{G}},s}} = R_{{\text{G}}} \frac{{d_{x} }}{{w_{x} }} - {\text{i}}\frac{1}{4}\frac{\lambda }{{d_{x} }}\eta_{0} \left[ {\ln \left( {\sec \phi } \right) + \frac{{\delta \sin^{4} \phi }}{{1 + \delta \cos^{4} \phi }}} \right]^{ - 1} ,$$

and

4b $$Z_{{\text{G,p}}} = R_{{\text{G}}} \frac{{d_{x} }}{{w_{x} }} + {\text{i}}\frac{{d_{x} }}{\lambda }\eta_{0} \left[ {\ln \left( {\csc \phi } \right) + \frac{{\delta \cos^{4} \phi }}{{1 + \delta \sin^{4} \phi }}} \right],$$

where

4c $$\delta = \frac{1}{{\sqrt {1 - \left( {{{d_{x} } \mathord{\left/ {\vphantom {{d_{x} } \lambda }} \right. \kern-\nulldelimiterspace} \lambda }} \right)^{2} } }} - 1, \, \phi = \frac{{\pi w_{x} }}{{2d_{x} }}$$

and *R*_G_ is the graphene impedance that we will describe in the following sub-section.

### Impedance relation

Based on (3) and (4), the extinction and impedance magnitudes (α_1D_ and R_G_) are interrelated. So, to estimate the extinction coefficient accurately, we must genuinely predict the impedance magnitude. As we mentioned earlier, we have recently developed^5^ exact plasmon–phonon dispersion relations for graphene placed on polar substrates, using dielectric function expanded by RPA (See Supplementary Information for ε_RPA_). Loss function, related to the Im{ε_RPA_}^−1^, directly relates to the graphene impedance. The behavior of the loss function depends on the intrinsic optical phonons and extrinsic S-POPs^5^, which means the impedance also depends on the numbers of the inherent optical phonons and extraneous vibrational modes. So the impedance takes its value based on the type of the underlying dielectric substrate. In other words, the graphene impedance relation linearly depends on the total number of optical phonons obtained by the Bose–Einstein distribution^22^,

5 $$N_{{{\text{phT}}}} = \sum\nolimits_{j} {N_{{{\text{phj}}}} } = \sum\nolimits_{j} {\frac{1}{{\exp \left( {{{\hbar \omega_{{{\text{phj}}}} } \mathord{\left/ {\vphantom {{\hbar \omega_{{{\text{phj}}}} } {k_{{\text{B}}} T}}} \right. \kern-\nulldelimiterspace} {k_{{\text{B}}} T}}} \right) - 1}},}$$

in which *N*_phj_ and *ћω*_phj_ stand for the number and energy of phonons of the *j-*th excited mode (*j* = op for the intrinsic phonons and 1, 2, 3 for the extrinsic S-POPs), ω_phj_ is the related radian frequency, *ћ* is the reduced Planck’s constant, and *k*_B_ and *T* are the Boltzmann constant and the ambient temperature, respectively. At low temperatures, the number of optical phonons is small enough not to participate in plasmon hybridization and damping. The phonon’s association starts to be significant as the temperature increases and, then, the impedance enhances due to the increased electron–phonon scattering. Therefore, the impedance directly links to the total number of phonons too. Hereafter, we work at room temperature, *T* = 300 K. Knowing all these, we introduce a new formula for the graphene impedance that is directly proportional to the loss factor and the total number of phonons,

6 $$R_{{\text{G}}} = - \beta d_{x}^{2} \eta_{0} {\text{Im}} \left( {\frac{1}{{\varepsilon_{{{\text{RPA}}}} }}} \right)N_{{p{\text{hT}} }} ,$$

in which ε_RPA_ follows (S9) and (S10) in Supplementary Information for non-polar and polar dielectric substrates, and *β* (m^−2^) is a fit parameter, providing a perfect match between the experimental and theoretical extinction data. The value of the fit parameter strongly depends on the underlying dielectric type — i.e., whether it is nonpolar or polar, and the number of POPs oscillations for the polar material. For the fit to be practicable, we need some reliable experimental data to compare. Hence, we introduce the fit parameter (*β*) solely for DLC and SiO_2_, for which experimental data are available^13^. In other words, the model is a semiempirical one that applies to any nonpolar/polar dielectric material for which experimental data are available. To arrive at (6), we carefully examined all parameters that the impedance depends on, using experimental results reported earlier^6,13^, whereas previous studies solely considered the dependency of the impedance on either the number of phonons^6^ or the loss parameter^13^. Yet, other groups^14,15^ modeled the impedance using the Drude model, ignoring the phonons’ effect that is particularly crucial for operating near or above the phonon frequencies. Hence, to the best of our knowledge, (6) is the most accurate and straightforward equation describing the impedance that can be used to predict the behavior of the graphene-based plasmonic devices operating below the mid-infrared wavelength of ~ 6.2 μm.

## Results and discussion

### Graphene on a non-polar dielectric

**Figure 2:**
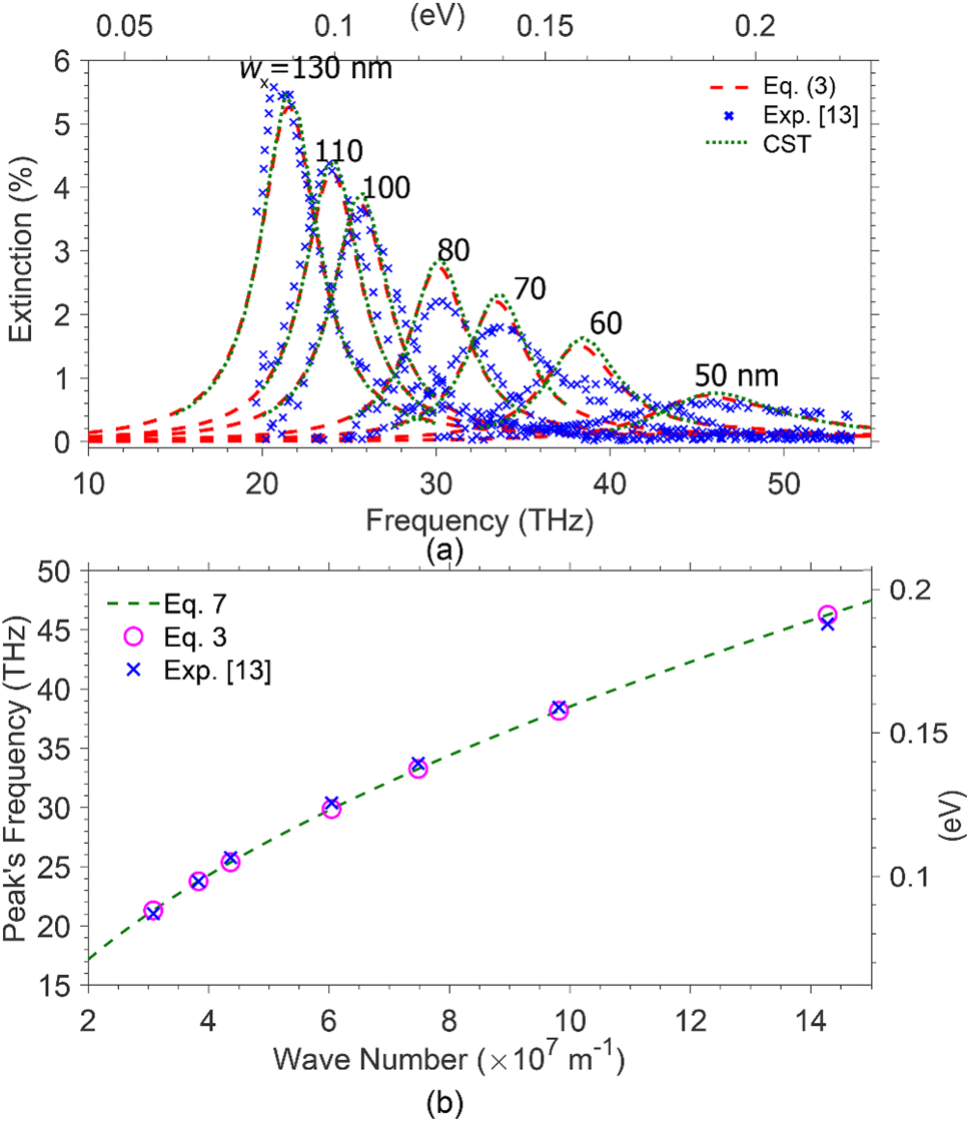
(**a**) Extinction spectra of graphene ribbons of widths 50 nm ≤ *w*_*x*_ ≤ 130 nm, pitches *d*_*x*_ = *2w*_*x*_ and *μ*_*C*_ = 0.3 eV on DLC substrate. The red dashes represent data obtained Eqs. (3) and (6) with *β* = 529 μm^−2^, to fit the experimental data^13^ (blue crosses). The data shown by the green dots are the results from CST simulations. (**b**) The plasmons peak’s frequency versus the wave vectors, *q*, obtained from (3) as shown 1(a) (open circles), from the experimental data^13^ (crosses), and Eq. (7) (dashes).

First, we considered arrays of graphene ribbons of widths (50 nm ≤ *w*_x_ ≤ 130 nm) and pitches *d*_x_ = 2*w*_x_ placed on a non-polar (DLC) substrate of relative permittivity ε_DLC_ = 5.7—i.e., similar structures used in an experimental study by^13^ —and calculated the extinction spectra, using (3) -(6) and (S9) with *β* = 5.29 × 10^14^ m^−2^, to have the best fit to the experimental data reported by^13^. The red dashes and blue cross signs (x) in Fig. 2a depict our numerical results and the experimental data^13^ for graphene ribbons of the given widths and chemical potentials, *μ*_C_ = 0.3 eV. Then, introducing graphene as a material with impedance (6) in the CST Microwave Studio software, we calculate the reflection, *R*, and absorption, *A*, coefficients for the array of graphene ribbons on the dielectric, which results in the extinction spectrum through (*A* + *R*), as shown by the green dots in Fig. 2a. For more information about using CST built-in BASIC interpreter (VBA Macro Language) for graphene as a material, see Supplementary Information. The same approach is also applicable to other commercial software. This figure demonstrates an excellent agreement between all three sets of data, confirming the correctness of our developed theory. Besides being a compact formula, Eq. (3) requires a much shorter time for calculating the extinction spectrum over a wide range of frequencies—i.e., less than a second—whereas CST simulation for the desired frequency range consumes a much longer time.

As shown in the figure, the extinction spectrum for each array, with given *w*_*x*_ and *d*_*x*_*,* exhibits a dominant resonance peak, decreasing as w_x_ (*d*_*x*_) becomes narrower. Moreover, the corresponding spectral width becomes thicker, experiencing a blueshift. One may attribute these to the increase in the damping of surface plasmons. Various scattering mechanisms like electron scattering by background ionized impurities, optical phonons, the edge of the arrays, and electron–hole generation within the single-particle excitation region may dampen the plasmons, becoming less probable as *w*_*x*_ increases. Background scattering highly depends on the fabrication process, and for a well-made graphene layer, the electron lifetime due to impurities takes the value of 85 fs^13^. The surface plasmons induced on a superstructure are affected by intrinsic phonons. They damp through emitting inherent phonons and enter the phonon emission (PE) continuum if their frequency becomes more significant than *ω*_op_ (i.e., the phonon’s frequency)^12,13^. For *ω* < *ω*_op,_ where the phonon emission is not considerable, the edge scattering, resulting in high-energy high-momentum intervalley phonon emission^2^, is the dominant effect among damping processes. So, plasmon peaks lessen as the width decreases—decreasing width equals heightening edge effects. In addition, plasmons fall within the interband single-particle excitation continuum and decay into electron–hole pairs at frequencies much higher than that of inherent optical phonons’. Our desired frequency range does not include electron–hole excitations*.*

In 1-D periodic arrays, the plasmons’ wavenumber *q*_pl_ ≈ π / (*w*_*x−*_*w*_0_), in which *w*_0_ ≈ 28 nm is the electrically inactive width of structured graphene^13^. The open circles and crosses ( ×), in Fig. 2b, show the dependence of the extinction peak’s frequency versus the surface plasmons wavenumber, *q*_pl_, obtained from (3) and the experimental data^13^, as shown in 2(a). The dashes represent the dispersion relation obtained for the similar graphene ribbons arrays on nonpolar substrates^5^,

7a $$\omega_{{{\text{pl}}}} = \sqrt {\frac{{e^{2} \mu_{{\text{C}}} }}{{2\pi \hbar^{2} \varepsilon_{0} \varepsilon_{{{\text{env}}}} }}q_{{{\text{pl}}}} + \gamma^{2} } ,$$

where *e* is the elementary charge, *ε*_0_ is the free-space permittivity and *ε*_env_ is the average dielectric constant of the upper and the lower media surrounding the graphene array, and

7b $$\gamma^{2} = \frac{{v_{{\text{F}}}^{2} q_{{{\text{pl}}}}^{2} }}{8 }\left[ {\exp \left( {{{q_{{{\text{pl}}}} } \mathord{\left/ {\vphantom {{q_{{{\text{pl}}}} } {q^{\prime}}}} \right. \kern-\nulldelimiterspace} {q^{\prime}}}} \right) - 1} \right]^{ - 1} ,$$

with *q′* = 2πε_0_ε_env_(ℏω_op_)^2^/*e*^2^*μ*_*C*_ as the plasmons wavenumber at the frequency *ω* = *ω*_op_, vanishing at *q*_pl_ ≪ *q′* —i.e., *γ*(*q*_pl_ ≪ *q*′) → 0. Figure 2b demonstrates how accurately the plasmonic peaks obey the dispersion relation.

**Figure 3:**
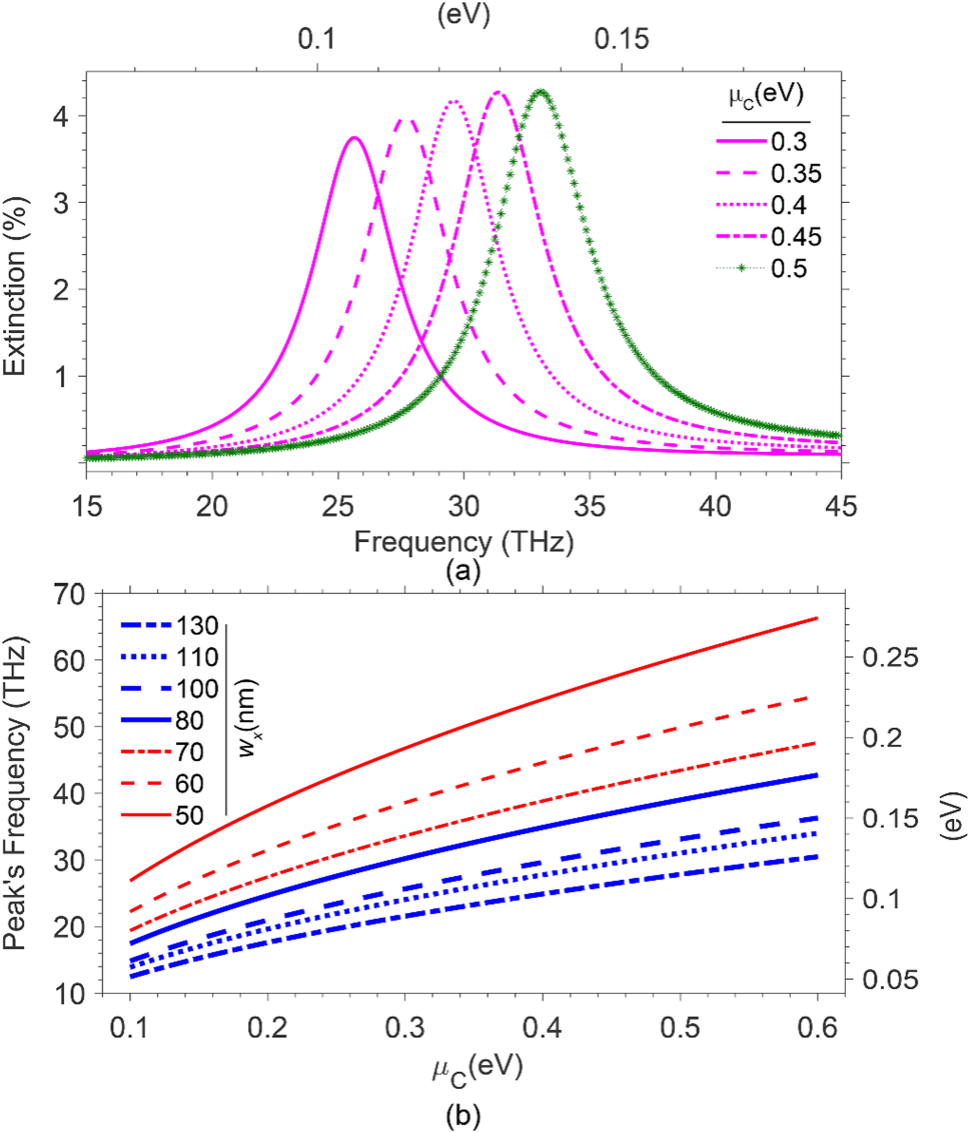
(**a**) Extinction spectra for the array of widths *w*_x_ = 100 nm, at different chemical potentials, 0.3–0.5 eV. (**b**) The resonance frequencies versus *μ*_*C*_ , for the arrays of widths *w*_*x*_ = 50–130 nm.

Another critical factor in determining the extinction peak frequency is the graphene’s chemical potential for a given array. According to (7a), $\omega_{pl} \propto \sqrt {\mu_{{\text{C}}} } ,$ conveying an increase in *μ*_*C*_ causes a blueshift in peak frequency. So, we have considered an array of widths *w*_*x*_ = 100 nm and calculated the extinction spectra while varying the chemical potential in the range of 0.3 eV ≤ *μ*_*C*_ ≤ 0.5 eV. Figure 3a illustrates the calculated spectra, using (3). Then, we plotted the extinction peak frequency versus the chemical potential for all seven arrays of Figs. 2 in 3b, demonstrating a more transparent presentation of the blueshifts caused by the increase in *μ*_*C*_. As seen from this figure, one can conclude that the narrower the width of the ribbons, the larger the blueshift caused by an increment in *μ*_C_. This figure gives us a piece of valuable information about how to choose a design parameter. Considering a CO_2_ laser of operating frequency ~ 28.5 THz (0.117 eV)^19^ as the light source, the best choice for *w*_x_ of the graphene ribbons with chemical potential in the range of *μ*_*C*_ ~ 0.3–0.4 eV would be 80–100 nm.

### Graphene on the polar dielectric

**Figure 4:**
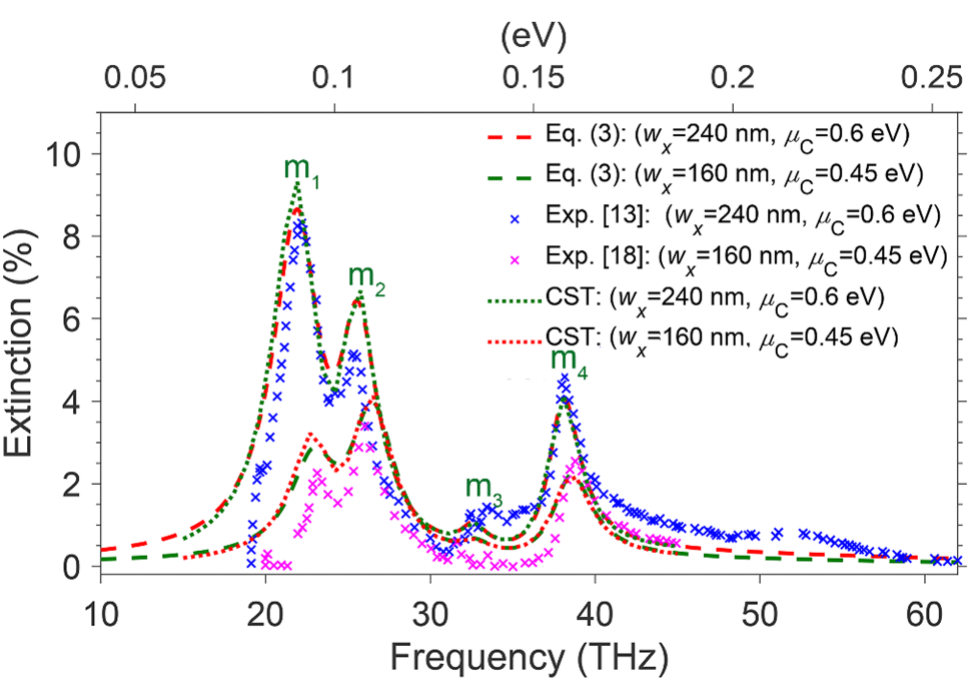
Extinction spectra for two sets of graphene ribbons (i.e., *w* = 240 (160) nm and *μ*_*C*_ = 0.6 (0.45) eV) placed on SiO_2_ with surface optical phonons of energies *ħω*_1, 2, 3_ = 100, 134, and 144.8 meV. The red (green) dashes show the corresponding results obtained from (3), the blue (magenta) crosses represent the experimental results reported by^13,18^, and the green (red) dots show the CST outcome, using (6). The labels *m*_1_* − m*_4_ denote the peaks of the four coupled phonons-plasmons modes.

Electrons in graphene placed on polar substrates are exposed to both intrinsic and extrinsic phonons. Besides the effects of intrinsic phonons mentioned in the previous subsection, extrinsic phonons cause mode splitting around their vibrational frequencies and turn single plasmonic mode into *n* + 1 coupled plasmon–phonon modes, where *n* is the number of the extrinsic vibrational frequencies^5^. Here, we consider two arrays of graphene ribbons of *w*_x_ = 240 and 160 nm and μ_C_ = 0.6 and 0.45 eV, placed on SiO_2_ slabs — i.e., a polar dielectric with *n* = 3 and *ħω*_1, 2, 3_ = 100 meV (24.63 THz), 134 meV (32.68 THz), and 144.8 meV (35.32 THz), just the same as those used in two independent experimental studies^13,19^. The red and green dashes in Fig. 4 represent the numerical results obtained from (3), fitted to the experimental results depicted by crosses (blue^13^ and magenta^19^, respectively), using the fit parameter *β* = 3.063 × 10^12^ m^−2^. The green and red dots in this figure depict the corresponding numerical results obtained by CST simulations, using Eq. (6) with the dispersion relations for the intrinsic and the three relevant extrinsic phonons modes we have already developed in^5^. Moreover, *N*_ph_ in (6) for this case is the total number of phonons provided for all vibrational frequencies.

The labels *m*_1_ − *m*_4_, seen in this figure, denote the peaks of the four coupled plasmons-phonons modes for both arrays, with center frequencies ω_*m*1_ < ω_1_, ω_1_ < ω_*m*2_ < ω_2_, ω_2_ < ω_*m*3_ < ω_3_ and ω_3_ < *ω*_*m*4_. A notable feature shown in this figure is the damped peak of the third coupled-mode denoted by *m*_3_*.* The third mode starts to rise at a frequency greater than *ω*_2_. However, the proximity of the S-POPs frequencies *ω*_2_ and *ω*_3_ prevent the third hybrid mode, *m*_3_, peaks significantly. In other words, the mode *m*_3_ is too weak to be practically identified as an exciting hybrid mode. Here again, the closeness of the results from our theoretical model to the experimental results confirms the accuracy of the developed model. Nonetheless, the minor deviation between the fit with two different experimental results could be due to a slight dependence of the fit parameter on *μ*_C*.*_

**Figure 5:**
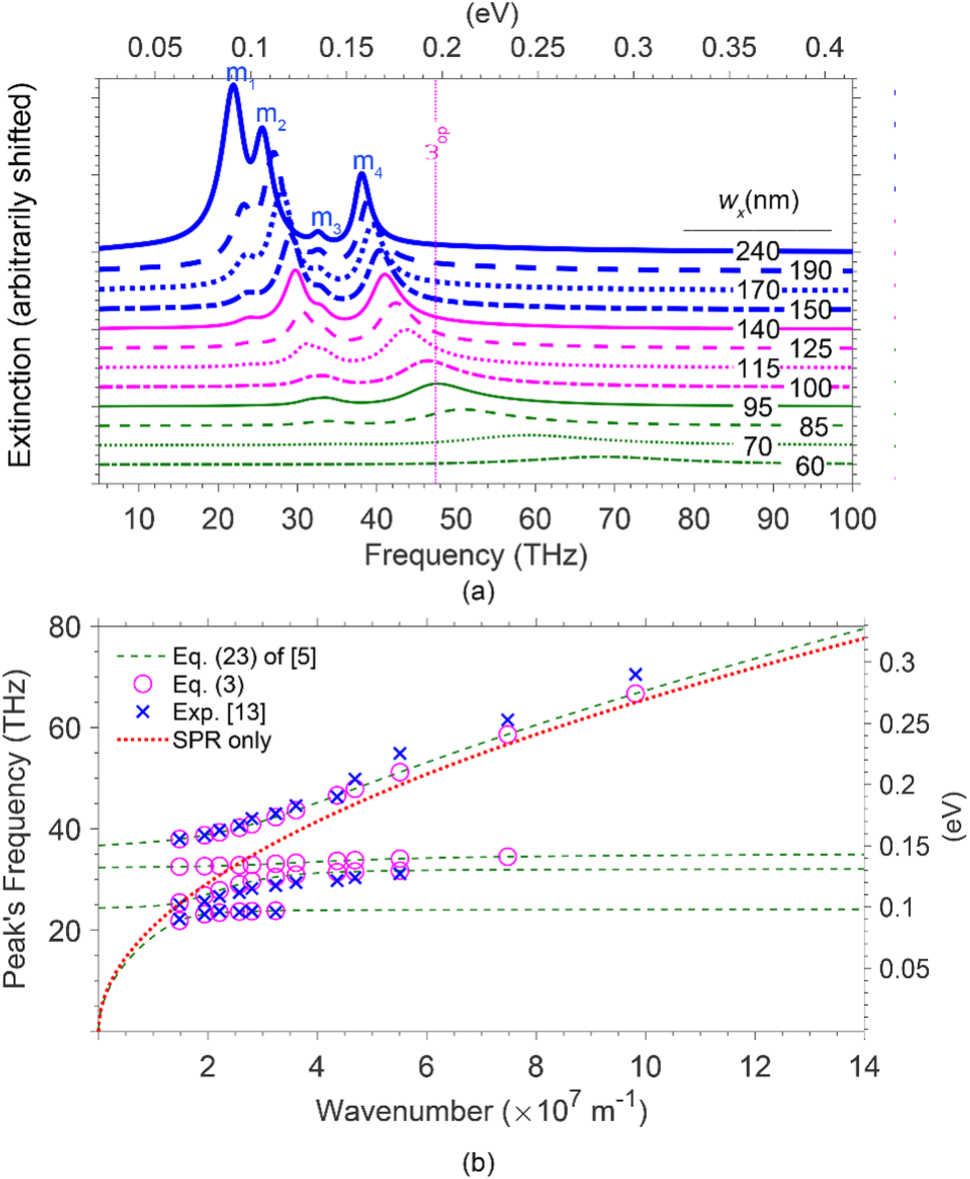
(**a**) Extinction spectra for graphene ribbons of different ribbons widths (60 ≤ *w* ≤ 240 nm) and *μ*_*C*_ = 0.6 eV placed on SiO_2_. These spectra are vertically displaced for clarity. The vertical dashed line indicates the graphene optical phonon frequency. The labels *m*_1_* − m*_4_ denote the peaks of the four coupled phonons-plasmons modes. (**b**) Peaks of the four coupled modes frequencies versus the plasmons’ wavevector, *q*, extracted from (a). The open circles represent the results obtained from (3), crosses depict the experimental results extracted from^13^, and the four dashed lines represent the modes dispersions calculated using (23) of^5^. The red dots depict the dispersion of the SPRs with no optical phonons present.

Next, we further compared the model with the experimental results of^13^, varying the widths of the graphene ribbons in the range of 60 ≤ *w*_*x*_ ≤ 240 nm and keeping the chemical potentials constant, *μ*_C_ = 0.6 eV. Then, we calculated the extinction spectra in the frequency range of 10–100 THz (0.041–0.41 eV) (Fig. 5a) and the dependencies of the corresponding peak’s frequency on the plasmons wavenumber, *q,* as depicted in Fig. 5b. The flat parts of the extinction spectra in Fig. 5a almost coincided, making them indistinguishable. Hence, to make them distinguishable, we shifted the vertical axis of each curve arbitrarily. The vertical dots in this figure denote the position of the inherent optical phonons frequency, ω_op_. As seen in this figure, four apparent futures are accompanying the reduction in *w*_*x*_, in agreement with the experimental results of^13^. Those features are (i) as *w*_*x*_ decreases, each resonance peak for a given array exhibits a blueshift that differs from one to another; (ii) unlike the other three modes, the blueshift exhibited by *ω*_m3_ is insignificant because of the closeness of *ω*_2_ and *ω*_3_, as mentioned earlier; (iii) the weight of the mode intensity transfers from m_1_ to m_2_ and then to m_4_, before the resonance peak for m_4_ reaches the onset of the PE continuum (ω_m4_ → *ω*_op_), beyond which it starts to damp through the emission inherent phonons (a similar phenomenon observed by^5^); (iv) the linewidth of m_4_ increases as ω_m4_ → *ω*_op_ and beyond, due to the damping through intrinsic optical phonon emission.

Figure 5b shows the dependencies of the extinction peaks frequencies on the plasmons wavenumber that inversely varies with the ribbons widths—i.e., *q*_pl_ ∝ (*w*_*x−*_*w*_0_) ^−1^. The open magenta circles and blue crosses represent the peak frequencies obtained from (3) and the experimental results of^13^, respectively. The four dashed curves show the dispersion curves obtained from (23) of^5^. Moreover, the red dots show the dispersion of the surface plasmons resonance (SPRs) on the graphene array ignoring the effects of the intrinsic and extrinsic optical phonons. A comparison of these data reveals the coupled modes peaks and SPR frequencies coincide far from splitting energies, where S-POPs have negligible effects, providing only electrons participate in the excitations. Hence, the hybridization of the plasmon–phonon prevails as the frequencies of hybrid modes and phonons are in proximity.

**Figure 6:**
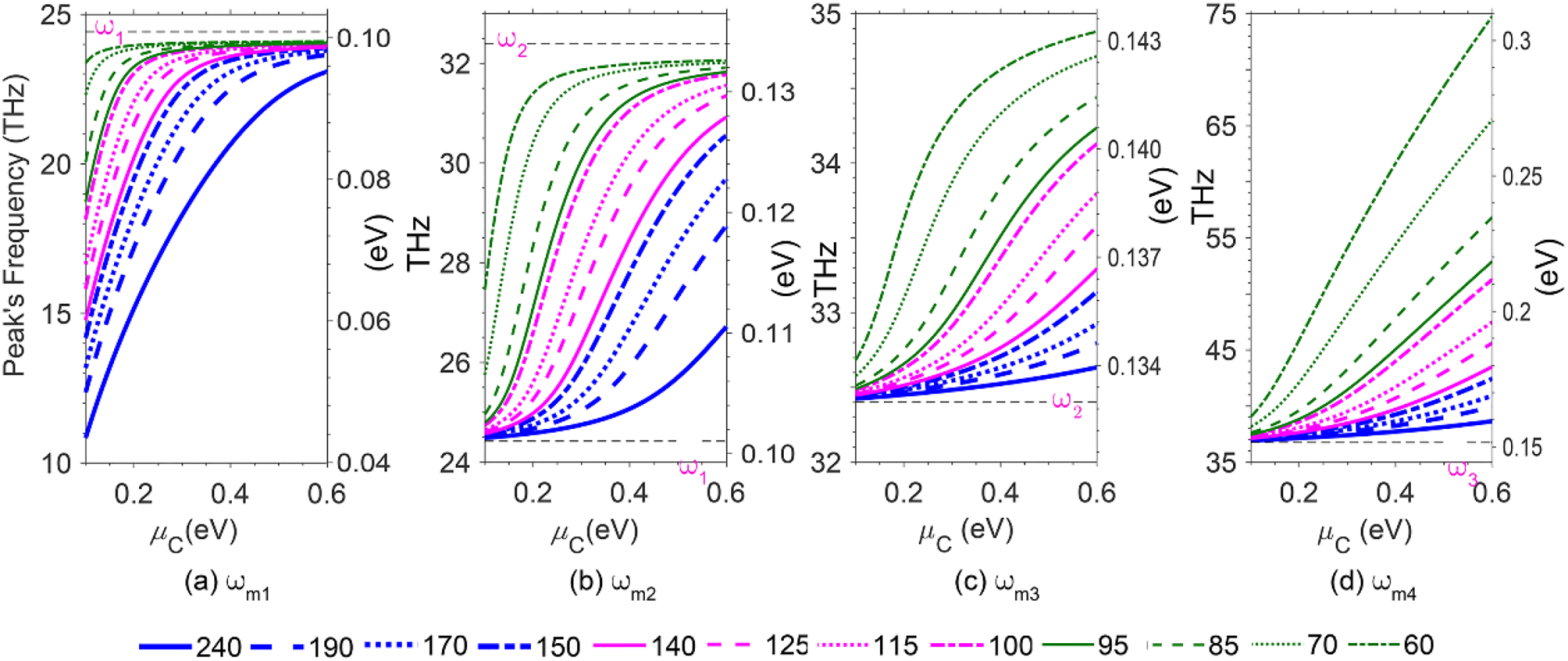
The peak frequency of the hybrid mode (**a**) *ω*_m1_, (**b**) *ω*_m2_, (**c**) *ω*_m3_, and (**d**) *ω*_m4_ versus chemical potential (*μ*_C_) for different ribbons widths. Horizontal dashes show the corresponding S-POPs vibrational frequencies, as indicated in each part.

Finally, we investigated the effects of the graphene chemical potential, *μ*_C_, on the center frequencies of the extinction peaks (ω_m1-4_), varying the ribbons' widths (see Fig. 6). The Horizontal dashes represent the vibrational frequencies of the corresponding S-POPs, as indicated in each part, showing the relations ω_*m*1_ < ω_1_, ω_1_ < ω_*m*2_ < ω_2_, ω_2_ < ω_m3_ < ω_3,_ and ω_3_ < *ω*_m4_ remain independent of the ribbons widths and chemical potentials. The proximity of ω_2_ and ω_3_ is why ω_m3_ is the least affected resonant frequency even by the extreme change in *μ*_C_ for the narrowest ribbons. The plots in this figure help a designer easily find the appropriate design parameters (*d* and *μ*_C_) to achieve the desired hybrid mode frequency. These plots also confirm the blueshift exhibited by each extinction peak as *w*_x_ decreases, for a given chemical potential, varying from one mode to another*.* Moreover, a common feature observed for the resonant peaks m_2_, m_3_, and m_4_ is not true for m_1_, that the narrower the ribbons in the array, the larger the blueshift exhibited by the resonant peak as *μ*_*C*_ increases. Furthermore, a variation in the chemical potential has influenced ω_m4_ the most for the narrowest ribbons.

**Figure 7:**
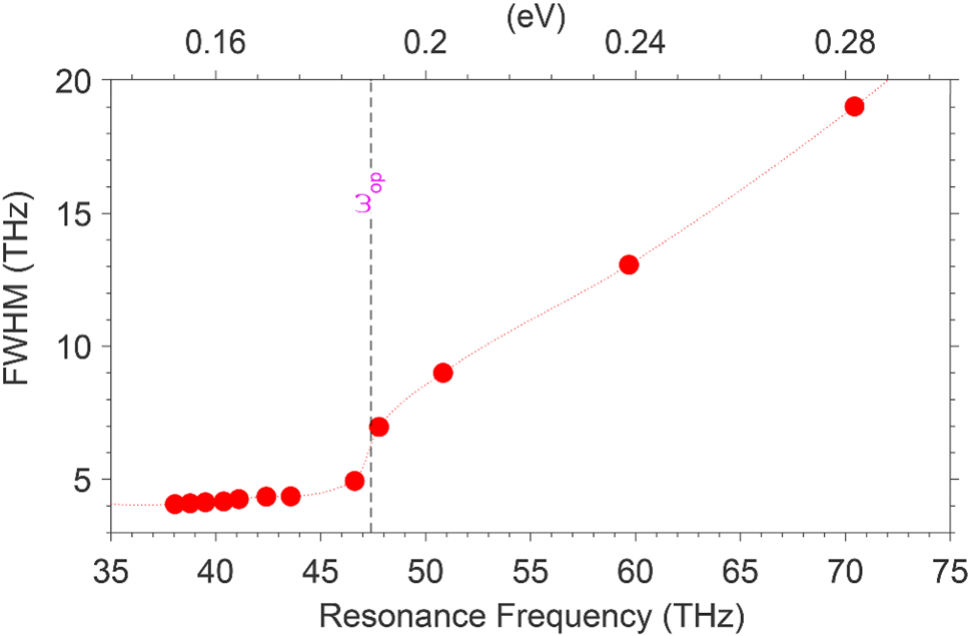
FWHM for m_4_ as a function of the corresponding plasmon resonance frequency extracted from Fig. 5a. The dashes show the locus of *ω*_op_.

By extracting full width at half maximum (FWHM) of the hybrid mode m_4_ in Fig. 5a and further observation into the inherent phonon damping, the impact of optical phonons at mode damping becomes clearer. Figure 7 depicts the extracted FWHM versus the resonance peak frequency. The vertical denotes the locus of *ω*_op_. It is evident that for peaks’ frequencies higher than *ω*_op_, the resonance peak damps more rapidly due to the inherent phonon emission.

## Conclusion

Utilizing the dielectric function obtained by random phase approximation and the number of optical phonons, we have modeled the resistance of graphene placed on a non-polar, DLC, and polar, SiO_2_, dielectric and then calculated extinction spectra, which match with the experiment perfectly. Our results demonstrate that the S-POPs convert a single pure plasmonic mode into multiple hybrid plasmon–phonon resonances, which have never been shown theoretically. Moreover, our presented model predicts the damping caused by optical phonon emissions. Our article provided a new formula of extinction based on the impedance boundary condition method, which the experimental data and CST simulation confirm.

## Supplementary Information

Supplementary Information.

## Data Availability

Data underlying the results presented in this paper are not publicly available at this time but may be obtained from the corresponding author upon reasonable request.
