# Supplementary material for: Semiempirical modeling of the effects of the intrinsic and extrinsic optical phonons on the performance of the graphene-based devices
Source: Sci Rep. 2022 Jun 21;12:10417. doi: 10.1038/s41598-022-14073-5 (PMC9213504; doi:10.1038/s41598-022-14073-5)
Supplement: Supplementary file 1 — Supplementary Information. [file 41598_2022_14073_MOESM1_ESM.docx]

**Supplementary:**

**Semiempirical Modeling of the Effects of the Intrinsic and Extrinsic Optical Phonons on the Performance of the Graphene-based Devices**

1. **Dielectric Function Obtained by Random Phase Approximation**

Here, we describe the modeling of the RPA expanded dielectric function briefly, fully explained in our earlier work [5]. In this approach, a single electron is exposed to a self-consistent field, including those induced by phonons and other electrons. If we consider electron-electron and electron-phonon interactions as the primary sources of scattering, the RPA expansion of the dielectric function, *ε*_RPA_, can be expressed by

$V_{\text{eff}}=\left(V_{\text{C}}+V_{\text{ph}}\right)/\varepsilon_{\text{RPA}}\left(q,\omega\right).$ (S1)

In which *V*_eff_ is the effective potential that an electron experiences in a weak many-body system,

$V_{\text{eff}}=\left(V_{C}+V_{\text{ph}}\right)/\left[\varepsilon_{\text{env}}-\left(V_{C}+V_{\text{ph}}\right)\Pi_{0}\right].$ (S2)

By comparing these equations, one can realize that

$\varepsilon_{\text{RPA}}\left(q,\omega\right)=\varepsilon_{\text{env}}-\left(V_{C}+V_{\text{ph}}\right)\Pi_{0}$ (S3)

where *ε*_env_ is the average of the dielectric constants of the upper and lower media surrounding the graphene, *V_C_ = e^2^/2qε_0_* is the Coulomb interaction between two electrons, in which *e, q,* and *ε*_0_ are the elementary charge, the electron wave number, and the free-space permittivity, respectively. *V*_ph_ = *v*^op^ + *v_λ_*^POP^ is the interaction between two electrons mediated by both intrinsic (*v*^op^) and extrinsic (*v_λ_*^POP^) optical phonons, introduced by

(S4)

and

(S5)

where λ is the phonon identifier, *g*^o^ **_q_**^p^_λ_^(POP)^ is the transition matrix elements related to electron-optical phonon interaction, introduces the phonon Green’s function. The identifier λ is omitted for intrinsic optical phonon. These parameters are obtained through,

(S6)

for inherent optical phonons, where ℏ is Planck’s constant, *D*_op_≈ 11 eV∙Å^−1^ is the optical phonon deformation potential, ρ_m_≈ 7.6⨯10^−7^ kg∙m^−2^ is graphene mass density, *ħω*_op_= 198 meV and τ_op_≈ 70 fs are the intrinsic optical phonon energy and lifetime, and *ω* represents the input optical signal frequency. For extraneous phonons, we have

(S7)

and is similar to (S6), in which *ω*_op_ and *τ*_op_ are replaced by the extrinsic phonons frequency, *ω*_λ_, and lifetime, *τ*_POP_.

Fröhlich coupling strength, , describes the magnitude of the polarization field of the phonon labeled λ. For more details about the values of the parameters, please see [5]. In (S3), Π_0_ is two-dimensional polarizability, which is introduced by,

(S8)

*μ*_C_ is the chemical potential, *τ*_pl_ is the plasmons lifetime, and *γ* is a *q*-dependent parameter to eliminate the deviations of dispersion at high *q*s. A full explanation of how these are introduced is presented in [5].

Using the above theory, we can obtain a complete description of the dielectric function of graphene on non-polar substrates as

(S9)

and

(S10)

for polar ones. Then, the loss function related to Im{*ε*_RPA_}^-1^ is readily computable.

1. **Graphene Material Definition in CST Software:**

‘#Language “WWB-COM”

Option Explicit

Dim SubstrateS As String, MaterialNameS As String

Const T = 273

Const kB = 8.6180e-5

Const FermiVelocity=1e6

Const LightSpeed=2.99792458e8

Const ElectronCharge=1.6021766208e-19

Const Permittivity=8.8541878128e-12

Const PlanckConstant=6.582119514323906e-16

Const delta_ph = 2 * kB * T

Const gamma_0 = 0.018

Const m = 9.10938356e-31

Const BackgroundRelaxationTime=85e-15

Const DeltaBackground=BackgroundRelaxationTime ^ -1

Const OpPhononRelaxationTime=70e-15

Const DefPotential=11e10

Const MassDensity=7.6e-7

Const OmegaOptical=196e-3/PlanckConstant

Dim ep_env As Double

Dim Substrate As String

Dim ChemicalPotential As Double

Dim Fmin As Double, Fmax As Double

Dim nSamples As Long

Dim OmegaSSP1 As Double, OmegaSSP2 As Double, OmegaSSP3 As Double

Dim ep_0 As Double, ep_i1 As Double, ep_i2 As Double, ep_inf As Double

Dim FSPP1 As Double, FSPP2 As Double, FSPP3 As Double

Dim SPhononRelaxationTime As Double

Dim ElectronRelaxationTime As Double, Electron_OptRelaxationTime As Double, Electron_SPP1RelaxationTime As Double, Electron_SPP2RelaxationTime As Double, EdgeRelaxationTime As Double

Dim Delta_e As Double, Delta_Opt As Double, Delta_SPP1 As Double, Delta_SPP2 As Double

Dim alfa1 As Double, alfa2 As Double, Imag_Self_Energy_op As Double, Imag_Self_Energy_sp1 As Double, Imag_Self_Energy_sp2 As Double

Dim Periodicity As Double, Filling As Double, RibbonWidth As Double, WaveNumber As Double

Dim Number_of_Phonon As Double

Dim OmegaMin As Double, OmegaMax As Double, Omega As Double, DeltaOmega As Double

Dim VCoulomb As Double

Dim ReVop As Double,ImVop As Double

Dim ReVSPP1 As Double, ImVSPP1 As Double, ReVSPP2 As Double, ImVSPP2 As Double, ReVSPP3 As Double, ImVSPP3 As Double

Dim RePolarizability As Double, ImPolarizability As Double

Dim ReDielectricFunc As Double, ImDielectricFunc As Double

Dim ReSigma As Double, ImSigma As Double

Dim ReImpedance As Double, ImImpedance As Double

Dim preFactor1 As Double, preFactor2 As Double, preFactor3 As Double, preFactor_v_op As Double, preFactor_VSPP1 As Double, preFactor_VSPP2 As Double, preFactor_VSPP3 As Double, preFactor_Pol As Double

Dim q As Double, Gamma As Double, Beta As Double

Dim i As Long

Dim SurfaceConductivityObj As Object

Sub Main

Const ListArray_Substrate = Array("SiO2", "DLC (diamond-like-carbon)")

Dim sListArray_Substrate() As String, j As Long

ReDim sListArray_Substrate(UBound(ListArray_Substrate))

For j = 0 To UBound(ListArray_Substrate)

sListArray_Substrate(j) = ListArray_Substrate(j)

Next

Begin Dialog UserDialog 600, 310, "Resistance Calculation", .DialogFunc

Text 0, 20, 200, 20, "Substrate:", .Text2, 1

DropListBox 210, 15, 310, 21, sListArray_Substrate(), .SubstrateT

Text 0, 55, 200, 20, "Chemical Potential [eV]:", .Text3, 1

TextBox 210, 50, 150, 21, .ChemicalPotentialT

Text 0, 90, 200, 20, "Periodicity [um]:", .Text4, 1

TextBox 210, 85, 150, 21, .PeriodicityT

Text 0, 125, 200, 20, "Filling Factor [ < 1]:", .Text5, 1

TextBox 210, 120, 150, 21, .FillingT

Text 0, 160, 200, 20, "Minimum Frequency [THz]:", .Text6, 1

TextBox 210, 155, 150, 21, .MinFrequencyT

Text 0, 195, 200, 20, "Maximum Frequency [THz]:", .Text7, 1

TextBox 210, 190, 150, 21, .MaxFrequencyT

Text 0, 230, 200, 20, "Number of Points:", .Text8, 1

TextBox 210, 225, 150, 21, .NumPointsT

PushButton 400, 265, 80, 30, "OK"

CancelButton 500, 265, 80, 30

End Dialog

Dim dlg As UserDialog

MaterialNameS = "Graphene"

dlg.ChemicalPotentialT = "0.5"

dlg.PeriodicityT = "0.5"

dlg.FillingT = "0.5"

dlg.MinFrequencyT = "5"

dlg.MaxFrequencyT = "50"

dlg.NumPointsT = "1000"

If (Dialog(dlg) = 0) Then

Exit All

End If

End Sub

Function DialogFunc%(DlgItem$, Action%, SuppValue%)

Select Case Action%

Case 2

Select Case DlgItem$

Case "OK"

Substrate = DlgText("SubstrateT")

ChemicalPotential = CDbl(DlgText("ChemicalPotentialT"))

Periodicity = CDbl(DlgText("PeriodicityT")) * 1e-6

Filling = CDbl(DlgText("FillingT"))

Fmin = CDbl(DlgText("MinFrequencyT")) * 1e12

Fmax = CDbl(DlgText("MaxFrequencyT")) * 1e12

nSamples = CDbl(DlgText("NumPointsT"))

Select Case Substrate

Case "SiO2"

OmegaSSP1 = 101e-3 / PlanckConstant

OmegaSSP2 = 134e-3 / PlanckConstant

OmegaSSP3 = 144.8e-3 / PlanckConstant

ep_0 = 3.7

ep_i1 = 3.5

ep_i2 = 3.4

ep_inf = 2.7

Number_of_Phonon = 3

Beta = (1.75e6 * Periodicity) ^ 2

Case "DLC (diamond-like-carbon)"

ep_0 = 5.7

Number_of_Phonon = 0

Beta = (2.3e7 * Periodicity) ^ 2

End Select

Periodicity = CDbl(DlgText("PeriodicityT")) * 1e-6

Filling = CDbl(DlgText("FillingT"))

ep_env = (1 + ep_0) / 2

If Number_of_Phonon <> 0 Then

FSPP1 = 1 / (2 * PI) * PlanckConstant * OmegaSSP1 * (1 / (ep_i1 + 1) - 1 / (ep_0 + 1))

FSPP2 = 1 / (2 * PI) * PlanckConstant * OmegaSSP2 * (1 / (ep_i2 + 1)- 1 / (ep_i1 + 1))

FSPP3 = 1 / (2 * PI) * PlanckConstant * OmegaSSP3 * (1 / (ep_inf + 1)- 1 / (ep_i2 + 1))

End If

Periodic_GPN_Resistance(Number_of_Phonon, ChemicalPotential, Fmin, Fmax, nSamples, OmegaSSP1, OmegaSSP2, OmegaSSP3, FSPP1, FSPP2, FSPP3, Periodicity, Filling)

End Select

End Select

End Function

Function Periodic_GPN_Resistance(Number_of_Phonon, ChemicalPotential, Fmin, Fmax, nSamples, OmegaSSP1, OmegaSSP2, OmegaSSP3, FSPP1, FSPP2, FSPP3, Periodicity, Filling) As Double

Dim N_SPP1 As Double, N_SPP2 As Double, N_SPP3 As Double, N_OP As Double, Phonon_Num As Double

N_SPP1 = 0

N_SPP2 = 0

OmegaMin = 2 * PI * Fmin

OmegaMax = 2 * PI * Fmax

DeltaOmega = (OmegaMax - OmegaMin) / (nSamples - 1)

RibbonWidth = Periodicity * Filling

WaveNumber = PI / (Filling * Periodicity - 0.028e-6)

SPhononRelaxationTime = 250e-15

SPhononRelaxationTime = SPhononRelaxationTime * Exp(-1 * WaveNumber / 4e7)

VCoulomb = ElectronCharge / (2 * WaveNumber * Permittivity)

preFactor1 = DefPotential ^ 2 * ElectronCharge /(MassDensity * FermiVelocity ^ 2)

preFactor2 = 2 * PI * ElectronCharge / (PlanckConstant * WaveNumber * Permittivity)

preFactor3 = ChemicalPotential * WaveNumber ^ 2 / (PI * PlanckConstant ^ 2)

q = 2 * PI * PlanckConstant ^ 2 * Permittivity * ep_env * OmegaOptical ^ 2 / (ElectronCharge * ChemicalPotential)

Gamma = 0.125 * FermiVelocity ^ 2 * WaveNumber ^ 2 * (Exp(q / WaveNumber) - 1) ^ -1

Set SurfaceConductivityObj = Result1DComplex("")

With Material

.Reset

.Name MaterialNameS

.Type "Lossy Metal"

.MaterialUnit "Frequency", "THz"

.SetTabulatedSurfaceImpedanceModel "Transparent"

.DispersiveFittingSchemeTabSI "Nth Order"

.MaximalOrderNthModelFitTabSI 10

.ErrorLimitNthModelFitTabSI 0.0001

.UseOnlyDataInSimFreqRangeNthModelTabSI True

For i = 0 To nSamples - 1

Delta_e = DeltaBackground

Omega = OmegaMin + i * DeltaOmega

Electron_OptRelaxationTime = PlanckConstant / Im_Self_Energy(gamma_0, Omega, OmegaOptical)

Delta_Opt = Electron_OptRelaxationTime ^ -1

Delta_e = Delta_e + Delta_Opt

preFactor_v_op = preFactor1 * Factor( Omega, OmegaOptical, OpPhononRelaxationTime)

N_OP = (Exp(PlanckConstant * OmegaOptical / (kB * T)) - 1) ^ -1

Phonon_Num = N_OP

ReVop = 0

ImVop = 0

If Number_of_Phonon <> 0 Then

N_SPP1 = (Exp(PlanckConstant * OmegaSSP1 / (kB * T)) - 1) ^ -1

Phonon_Num = Phonon_Num + N_SPP1

preFactor_VSPP1 = preFactor2 * OmegaSSP1 * Factor(Omega, OmegaSSP1, SPhononRelaxationTime)

ReVSPP1 = preFactor_VSPP1 * FSPP1 * (Omega^2-OmegaSSP1^2 - (1/SPhononRelaxationTime)^2)

ImVSPP1 = -preFactor_VSPP1 * FSPP1 * (2*Omega/SPhononRelaxationTime)

N_SPP2 = (Exp(PlanckConstant * OmegaSSP2 / (kB * T)) - 1) ^ -1

Phonon_Num = Phonon_Num + N_SPP2

preFactor_VSPP2 = preFactor2 * OmegaSSP2 * Factor(Omega, OmegaSSP2, SPhononRelaxationTime)

ReVSPP2 = preFactor_VSPP2 * FSPP2 * (Omega ^ 2 - OmegaSSP2 ^ 2 - (1 / SPhononRelaxationTime) ^ 2)

ImVSPP2 = -preFactor_VSPP2 * FSPP2 * (2 * Omega / SPhononRelaxationTime)

N_SPP3 = (Exp(PlanckConstant * OmegaSSP3 / (kB * T)) - 1) ^ -1

Phonon_Num = Phonon_Num + N_SPP3

preFactor_VSPP3 = preFactor2 * OmegaSSP3 * Factor(Omega, OmegaSSP3, SPhononRelaxationTime)

ReVSPP3 = preFactor_VSPP3 * FSPP3 * (Omega ^ 2 - OmegaSSP3 ^ 2 - (1 / SPhononRelaxationTime) ^ 2)

ImVSPP3 = -preFactor_VSPP3 * FSPP3 * (2 * Omega / SPhononRelaxationTime)

End If

ElectronRelaxationTime = Delta_e ^ -1

preFactor_Pol = preFactor3 * 1 / ((Omega ^ 2 - (1 / ElectronRelaxationTime)^2 - Gamma) ^ 2 + (2 * Omega / ElectronRelaxationTime) ^ 2)

RePolarizability = preFactor_Pol * (Omega ^ 2 - (1/ ElectronRelaxationTime) ^ 2 - Gamma)

ImPolarizability = -preFactor_Pol * (2 * Omega / ElectronRelaxationTime)

ReDielectricFunc = ep_env - (VCoulomb + 1 * ReVop * (Omega ^ 2 / WaveNumber ^ 2 - FermiVelocity ^ 2) + 1 * ReVSPP1 + 1 * ReVSPP2 + 1 * ReVSPP3) * RePolarizability + ( 1 * ImVop * (Omega ^ 2 / WaveNumber ^ 2 - FermiVelocity ^ 2) + 1 * ImVSPP1+ 1 * ImVSPP2+ 1 * ImVSPP3)*ImPolarizability

ImDielectricFunc = -(VCoulomb+ 1 * ReVop * (Omega ^ 2 / WaveNumber ^ 2 - FermiVelocity ^ 2) + 1 * ReVSPP1 + 1 * ReVSPP2 + 1 * ReVSPP3) * ImPolarizability - ( 1 * ImVop * (Omega ^ 2 / WaveNumber ^ 2 - FermiVelocity ^ 2) + 1 * ImVSPP1 + 1 * ImVSPP2+ 1 * ImVSPP3) * RePolarizability

ReImpedance = (Phonon_Num / (Permittivity * LightSpeed)) * Beta * ImDielectricFunc / (ReDielectricFunc ^ 2 + ImDielectricFunc ^ 2)

ImImpedance = 0

.AddTabulatedSurfaceImpedanceFittingValue CStr(Omega / (2 * PI * 1e12)), CStr(ReImpedance), CStr(ImImpedance), CStr(1.0)

Next

.Colour 0.2, 0.2, 0.2

.Create

End With

End Function

Function Im_Self_Energy(int_Factor As Double, Omega As Double, Omega_ph As Double) As Double

Im_Self_Energy = int_Factor * Abs(PlanckConstant * Omega + PlanckConstant * Omega_ph + ChemicalPotential) * (Sigmoid((PlanckConstant * Omega - PlanckConstant * Omega_ph) / delta_ph) + Sigmoid((-PlanckConstant * Omega - PlanckConstant * Omega_ph) / delta_ph) + 2)

End Function

Function Factor( Omega As Double, Omega_ph As Double, Relaxation_Time As Double) As Double

Factor = 1 / ((Omega ^ 2 - Omega_ph ^ 2 - (1 / Relaxation_Time) ^ 2) ^ 2 + (2 * Omega / Relaxation_Time) ^ 2)

End Function

Function Sigmoid(x)

Sigmoid = 2 / (1 + Exp(-5 * PI * x / 6)) - 1

End Function
